# Enhancing FBG Sensing in the Industrial Application by Optimizing the Grating Parameters Based on NSGA-II

**DOI:** 10.3390/s22218203

**Published:** 2022-10-26

**Authors:** Yasser Elsayed, Hossam A. Gabbar

**Affiliations:** Faculty of Engineering and Applied Science, Ontario Tech University, 2000 Simcoe St. North, Oshawa, ON L1G0C5, Canada

**Keywords:** FBG sensors, grating parameters, NSGA-II, Fiber Bragg Grating, reflectivity, grating length, grating bandwidth

## Abstract

Fiber Bragg grating (FBG) technology has shown a mutation in developing fiber optic-based sensors because of their tiny size, high dielectric strength, distributed sensing, and immunity to high voltage and magnetic field interference. Therefore, FBG sensors significantly improve performance and accuracy in the world of measurements. The reflectivity and bandwidth are the main parameters that can dramatically affect the sensing performance and accuracy. Each industrial application has its requirements regarding the reflectivity and bandwidth of the reflected wavelength. Optimizing such problems with multi-objective functions that might t with each other based on applications’ needs is a big challenge. Therefore, this paper presents an optimization method based on the nondominated sorting genetic algorithm II (NSGA-II), aiming at determining the optimum grating parameters to suit applications’ needs. To sum up, the optimization process aims to convert industrial applications’ requirements, including bandwidth and reflectivity, into the manufacturing setting of FBG sensors, including grating length and modulation refractive index. The method has been implemented using MATLAB and validated with other research work in the literature. Results proved the capability of the new way to determine the optimum grating parameters for fulfilling application requirements.

## 1. Introduction

In Canada, Ken Hill discovered FBG in 1978 at a research communication center [1]. The concept of FBG is based on the change in the refractive index of the core of FBG due to absorbing ultraviolet light [2]. Since FBG was invented, FBG sensors have gained their potential in the optical sensing world due to their built-in pros such as their tiny size, low cost, immunity to high voltage, magnetic field, chemical, and electromagnetic interference, in addition to their accuracy and sensitivity [3]. On top of that, they provide the distribution or multiplexed sensing capability, which covers long distances and wide surface area. On the other hand, the conventional sensors cannot offer these unique features of the FBG sensors [4]. Therefore, FBG sensors have been utilized in various medical, civil, electrical, mechanical, aerospace, maritime, oil and gas, military applications, renewable energy and battery charging applications [5]. In addition, they have shown standard sensing for most physical signals, such as temperature displacement, strain, stress, and pressure [6,7].

The FBG is constructed inside the fiber ultraviolet light by making a periodic perturbation in the refractive index in the fiber’s core due to high-intensity exposure [8]. A brag wavelength has been yielded from the band rejection constructed by the grating zone’s constructive interference of the reflected wavelengths. That Bragg wavelength depends on the grating parameters, including grating length, refractive index, and the grating period. Therefore, changing these parameters due to exposure to physical signals such as pressure, strain, and temperature will change the yielded bragging wavelength, which is the key idea behind using FBG in sensing [9]. Because FBG sensing performance depends on the grating parameters, and the reflected wave from FBG is characterized mainly by the reflectivity and bandwidth, optimizing grating parameters is crucial for optimizing the sensing accuracy and getting the desired bandwidth and reflectivity that fulfills the applications’ requirements. These requirements are not the same for all industrial applications. For example, most industrial applications require narrow bandwidth, while some other applications, such as oil and gas, require a wide range of bandwidth [10]. In addition, the multiplexing feature of FBG enables measuring a wide-scale range of parameters, especially in the construction industry, oil and gas applications, and tracking the power in PV power systems [11]. Therefore, the bandwidth and reflectivity characteristics of the reflected wave of FBG are critical factors in multiplexing more sensing points with different wavelengths [12]. In partial shading PV application, temperature, and irradiance of an array of modules need to be monitored. So the multiplexing and distributing sensing will be beneficial for such applications [13]. To sum up, bandwidth, reflectivity, and sidelobe are the most critical parameters that need to be optimized to enhance the performance of the sensing capabilities of the FBG and fulfill the different applications’ needs. In addition, some sidelobes are there with the reflected signal at the brag wavelength, which is undesirable in sensing applications [14]. The problem is how to satisfy all applications’ needs when sometimes there is a conflict between these requirements. For instance, some require minimizing the bandwidth and maximizing the reflectivity, while other applications require maximizing the bandwidth and reflectivity. In conclusion, it is a multi-objective optimization problem in which the bandwidth and reflectivity are the optimized variables. Moreover, the grating parameters, including the grating length and modulation index, are the control variables considering the conflict between these objectives for some applications, as noted by surveying a couple of studies [15,16]. These studies reported a positive correlation between the reflectivity and the modulation index on one hand and grating length on the other hand. On the other hand, they showed a negative correlation between the bandwidth and grating length, while it is positively correlated with index modulation. To recap and highlight the problem for which this paper presents a solution, this paper aims to determine the optimum grating parameters, including the grating length, modulation index, and grating period, that realize the optimum bandwidth and reflectivity to suit every industrial application’s needs. For example, the optimization solution proposed by this research provides the industry with a tool to determine the optimum FBG grating parameters for their applications.

Before designing the proposed solution, literature has been searched and reviewed to fetch the research contributions for solving the problem above. As a result, some studies worked on determining the grating parameters for obtaining the maximum reflectivity and the minimum sidelobes intensity. Moreover, other studies determined the relation between bandwidth and reflectivity and the grating parameters [15,16]. These studies contributed some simulation and experimental work for reporting the bandwidth, sidelobe, and reflectivity at different values of grating length and index modulation for determining the optimum bandwidth and reflectivity. However, these research works did not contribute to determining the optimum values for different requirements of industrial applications. They consider a specific range of grating length from 0 to 10 mm and index modulation from 0.0005 to 0.002. Nevertheless, some other studies can be used to validate the results obtained by the proposed optimization method in the present study. Moving to the proposed solution in this study, this paper presents an optimization method based on the nondominated sorting genetic algorithm II (NSGA-II) for solving the current multi-objective optimization problem that has some contradictions in its objectives. This method has been selected because it can apply the Pareto optimization concept and determine the Pareto front that can fetch superior solutions compared with other solutions in the search space. At the same time, these solutions are non-dominant to other objectives that conflict with them. The concept of nondominated sorting is that Pareto dominance is utilized to sort the population. The process commences by moving the first-ranked individuals of the non-dominating members from the initial population to the first front. Then the remaining individuals are then ordered based on the non-dominating sorting procedure to determine the second front. The process continues, and all populations are sorted and categorized based on their ranks on their equivalent fronts [17]. Surveying literature on the contributions of the NSGA-II method to solve multi-objective problems, it is found that there are research works that utilize NSGA-II. For example, the assignment problem has been solved by using NSGA-II [18], in which N tasks are optimally assigned to N agents to maximize the performance of the task and minimize the total cost of achieving the tasks. In addition, the allocation problem has been optimized using NSGA-II, in which activities are optimally allocated between resources to maximize activities that can be handled by limited resources [19]. Furthermore, the most known traveling salesman problem has been optimized by using NSGA-II to find out the shortest path the salesman can take to visit a city and return to the starting point [20]. Moreover, the scheduling problem has been optimized using NSGA-II to determine the optimum sequence of processing of N jobs by M machines aiming to optimize the total flow time, waiting time, service time, and make span [21]. Therefore, in the end, it comes up with the Pareto front that offers the optimum values of grating parameters for the manufacturing decision makers to select the grating length, grating period, and index modulation by which they can obtain the bandwidth and reflectivity that suit their applications’ requirements. The obtained results are compared to those other studies that yielded the same input parameters for verifying the proposed methods [15,16]. In addition, Thereby, the proposed method’s performance has been validated, the same scenarios are tested using another standard optimization method, such as a genetic algorithm, and a comparison has been conducted. The paper is organized as follows: Section 2 presents the background and the parameters of FBG sensors. Section 3 describes the methodology of the proposed optimization method. Section 4 demonstrates the results. Ultimately, Section 5 concludes the work.

## 2. Background

**Figure 1:**
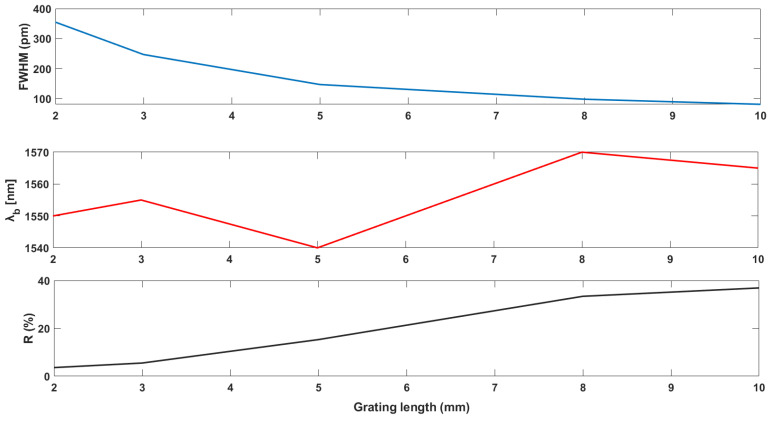
The effect of changing grating parameters on reflectivity and bandwidth [22].

Fulfilling the aim of this research, which is controlling the bandwidth and the power of the reflected wave of FBG by fetching the optimum grating parameters, it is important from the beginning to show the correlation between the control variables and the prospected optimized variables. Therefore, Figure 1 represents data obtained from one study in the literature showing the resulting bandwidth and reflected power of five FBG sensors with different grating parameters [22]. It depicts the effects of changing the grating parameters on the reflectivity and bandwidth. The variable R, the percentage of the reflected power, is positively proportional to the grating length. In contrast, the bandwidth represented by the full width at half maximum (FWHM) is negatively correlated. In addition, it is obviously shown how the base wavelength of the reflected wave is shifted by changing the grating length.

The reflected power and bandwidth of the reflected signal wave be mathematically represented as a function of the grating parameters as a relation between inputs and outputs. The inputs in this context are the grating length, grating period, and refractive modulation index, and the outputs are the reflected power and the bandwidth of the reflected wave. The reflection occurs at and only at the grating condition realized as in Equation (1) tells that the base wavelength of the reflected wave depends on the changing in the grating period and the refraction index.

(1) $$\lambda_{B}=2\;\Delta \;n_{eff}$$

where

$\lambda_{B}$: the Bragg base wavelength

Δ: the grating period

$n_{eff}\;$: the refractive index

For deriving a relation between bandwidth, reflectivity, sidelobe and grating length, and the change in refractive index. The bandwidth can be determined by Equation (2), which shows the bandwidth depends on the grating parameters, such as the variation in the refractive index [22].

(2) $$\Delta \lambda =\left[\frac{2\delta n_{0}\;\eta \;}{ᴫ}\right]$$

where

$\delta n_{0}$: the variation in the refractive index

η: the fraction of power in the core

In addition, the reflected and transmitted power can be determined by Equations (3) and (4) that also show the reflectivity depends on the grating parameters as follows [22]:

(3) $$P_{B}\left(\lambda \right)=\frac{{\sin h}^{2}\left(\eta \left(V\right)\delta n_{0}\sqrt{1-{Г}^{2}}\frac{N\Delta }{\lambda }\right)}{{\cos h}^{2}\left(\eta \left(V\right)\delta n_{0}\sqrt{1-{Г}^{2}}\frac{N\Delta }{\lambda }\right)-{Г}^{2}}$$

(4) $$Г\left(\lambda \right)=\frac{1}{\eta \left(V\right)\delta n_{0}}\left[\frac{\lambda }{\lambda_{B}}-1\right]$$

where

Δ: the grating periods

*V*: the fringe visibility

$L_{g}=N\Delta$: the grating length

Using the above mathematical equations makes it reasonable to analyze the correlation between these input and output parameters. 

**Figure 2:**
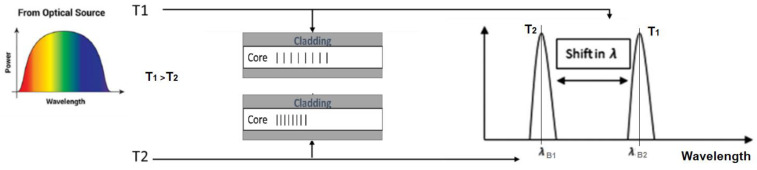
A conceptual example showing the effects of changing temperature on the base wavelength.

Figure 2 depicts a conceptual scenario showing how the FBG temperature sensor works. When an optical source indicated on the left of the figure is applied to the FBG sensor with a structure and grating shown in the middle of the figure that changes by increasing the applied temperature based on the coupled mode theory, which says the wavelength of Bragg depends on the effective refractive index and the grating period and represented mathematically in Equations (1)–(4) [22]. Therefore, for every change that occurred to the measured physical signal, there was an equivalent change that happened to the refractive index of the core of FBG, which, in turn, caused a shift in the base wavelength of the reflected wave. Figure 2 shows on the right the change in the base wavelength from $\lambda_{B1}\;$to $\lambda_{B2}$ due to changing the temperature from $T_{1}$ to $T_{2}$. 

In addition, optimizing a problem with multi-objective functions requires a suitable algorithm for determining the grating parameters that lead to optimum bandwidth and reflectivity of the reflected wave that fulfills applications’ needs. The following section presents the methodology and logic flow of the proposed optimization method to solve this multi-objective problem. 

## 3. Materials and Methods

The problem for whom this paper proposed a novel solution has multi-objective functions, which are maximizing the reflectivity, minimizing or maximizing the bandwidth based on the applications’ needs, and minimizing the sidelobe. Moreover, the control variables are the grating length, grating period, and the change in refractive index. In general, multi-objective functions can be formulated by determining *k* decisions that satisfy *n* objective functions and comply with m constraints as follows [23]:

(5) $$\min/\max f\left(x\right)=(f_{1}\left(x\right),f_{1}\left(x\right),f_{3}\left(x\right),\;\ldots \ldots ,\;f_{n}\left(x\right)$$

(6) $$E\left(x\right)=\left(e_{1}\left(x\right),e_{1}\left(x\right),e_{3}\left(x\right),\;\ldots \ldots ,\;e_{m}\left(x\right)\right)\leq 0$$

(7) $$X=\left(x_{1},x_{1},x_{3},\;\ldots \ldots ,\;x_{k}\right)\;\epsilon \;X$$

(8) $$Y=\left(y_{1},y_{1},y_{3},\;\ldots \ldots ,\;y_{n}\right)\;\epsilon \;Y$$

Subject to the following constraints

(9) $$h_{i}\left(x\right)\leq 0;\;\mathrm{for}\;i=1,2,3,\ldots \ldots ,p$$

(10) $$g_{j}\left(x\right)=0;\;\mathrm{for}\;j=1,2,3,\ldots \ldots ,u$$

The above equations formulate the optimization problem of multi-criterion decision making. In Equation (5), Equation (5) represents the set of solutions for minimizing or maximizing n objective functions 6ns above represents the set of restrictions or constraints. In addition, Equation (7) presents the set of the determined decisions, *X*. In addition, Equation (9) represents the space of objective *Y*. In addition, Equation (9) shows an example of inequality constraints. In contrast, Equation (10) presents the equality constraints. Solving this optimization problem yields Pareto-optimal solution that represents the most suitable region A where no region B is more feasible so that it could minimize or reduce some objectives and maximize or increase at least one other goal [23,24]. By projecting that concept on the current problem for whom this paper presents a solution, the objective functions might conflict, given that increasing the bandwidth does not go with increasing the reflectivity. Therefore, the appropriate solution for such a problem should apply Pareto optimization concepts, which is the mathematical optimization of two or more objective functions simultaneously without degrading the other functions [24]. The mathematical representation of a multi-objective criteria problem can be represented as follows:

(11) $$\mathrm{Min}|\mathrm{Max}\{f_{1}\left(\bar{x}\right),f_{2}\left(\bar{x}\right),f_{3}\left(\bar{x}\right),\ldots ,f_{k}(\bar{x})\}\;S.t\;\bar{x}\;\in X$$

With k≥2 is the minimum number of the objectives, and set *X* is the feasible set of vectors of decisions. In addition, the vector of the objective function can be formulated as follows:

(12) $$\vec{f}:X\overset{yields}{\to }\;R^{k},\;\vec{f}\;\left(\vec{x}\right)={\left(f_{1}\left(\vec{x}\right),f_{2}\left(\vec{x}\right),\ldots ,\;f_{k}\left(\vec{x}\right)\right)}^{T}$$

$\vec{x}\in X\;$is a feasible solution or decision and $\vec{f}\;\left(\vec{x}\right)$ is called an objective. 

In Pareto optimization, the target is to find a set of Pareto-optimal solutions that satisfy the condition that, for all points in the variable space, there is no other point that can give a less than or equal and at the same time it can give at least one value less than that is given by the Pareto-optimal point. That can be formulated mathematically as follows:

$$\mathrm{Pareto}\;\mathrm{optimal}:a\;\mathrm{point}\;X^{*\;}\in S\;\mathrm{is}\;a\;\mathrm{pareto}\;\mathrm{optimal}\;\mathrm{only}\;\mathrm{and}\;\mathrm{only}\;\mathrm{if}\;\;\;\mathrm{there}\;\mathrm{does}\;\mathrm{not}\;\mathrm{exist}\;\mathrm{another}\;\mathrm{point}\;X\;\in S\;\mathrm{such}\;\mathrm{that}\;f_{a}(X)\;\;\leq f_{a}(X^{*})\forall \;i\;\mathrm{and}\;f_{a}(X)<f_{a}(X^{*})\;\mathrm{for}\;\mathrm{at}\;\mathrm{least}\;\mathrm{one}\;i\mathrm{there}\;\mathrm{does}\;\mathrm{not}\;\mathrm{exist}\;\mathrm{another}\;\mathrm{point}\;X\;\in S\;\mathrm{such}\;\mathrm{that}\;f_{a}\left(X\right)\leq f_{a}\left(X^{*}\right)\forall \;i\;\mathrm{and}\;f_{a}\left(X\right)<f_{a}\left(X^{*}\right)\;\mathrm{for}\;\mathrm{at}\;\mathrm{least}\;\mathrm{one}\;i$$

The following section presents the proposed optimization method’s design by projecting the Pareto optimization on the problem of this study by using NSGA-II methods to determine the grating parameters for optimum bandwidth and reflectivity.

## 4. System Design

**Figure 3:**
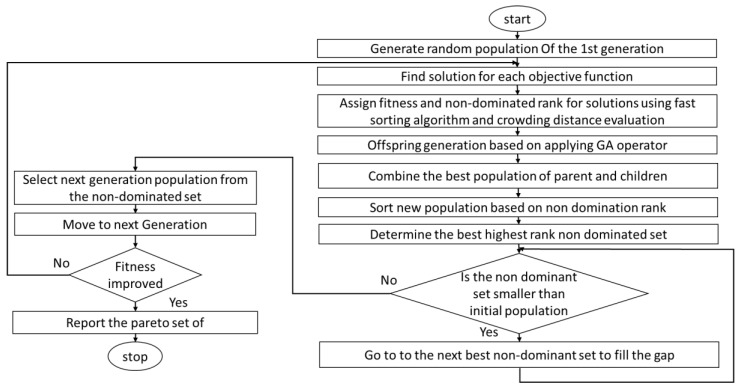
The nondominated rank-based sorting genetic algorithm (NSGA).

Based on the mathematical equations presented in the previous section, the reflected power of FBG and the bandwidth as objective functions can be modeled as a function of the grating length, grating period, and the change of refractive index. Moreover, from there, NSGA-II has been chosen for determining the Pareto front and calculating the optimum value of the control variable, which are grating length, grating period, and refractive index, for reaching the optimum values of the objective functions, which are the bandwidth and reflectivity with minimized sidelobe. Figure 3 charts the logic flow of NSGA-II. It starts with setting the initial parameters such as population size, number of generations, and initial populations of the grating parameters. Then it calculates each individual’s fitness in the initial population by calculating the bandwidth and reflectivity based on Equations (2)–(4). Then it assigns fitness and nondominated rank for each individual. Next, it sorts based on the level of nondomination calculation and the crowding distance evaluation. The next step is to apply GA operators such as mutation and crossover operators. Then it combines the population of the selected individual from the last generation based on the nondominated ranking and fitness along with the individuals yielded by the GA operators, i.e., crossover and mutation. After it selects the best nondominated individuals, it compares them with the initial population to check feasibility. If they are feasible solutions, they are chosen to be with the next generation; otherwise, it keeps calculating nondominated answers and ranking. Then it checks the termination condition, such as reaching the maximum number of iterations, to decide either to terminate or go back to repeating the process. Furthermore, RNSGA-II is a modified NSGA-II that enables setting reference values of the objectives for determining solutions that give the nearest to these references. The RNSGA-II has been utilized as an advanced method to help decision makers get the closest solutions to their preferences. Following this logic, the NSGA-II and RNSGA-II gain many advantages compared to GA and all other evolution algorithms. For example, because it is based on sorting the ranked nondominated solution, it becomes closer to the Pareto front solutions. By using the crowding distance technique, the algorithm has significant diversity in choosing the populations. Finally, it utilizes the elitist method to make sure of transferring the best solutions from one generation to the next. To prove the superiority of the selected method, NSGA-II, the optimization problem has been solved at the same conditions, and values of all parameters using G comparison have been conducted to validate the performance parameters.

## 5. Results

For testing the proposed optimization method, two methods are proposed for solving the optimization problem based on NSGA-II and RNSGA-II. In addition, one way is used for validation, which is GA. For each test case, there are two scenarios will be tested. The first scenario is when an industrial application requires increasing the reflectivity and narrowing the bandwidth, which is the case with most applications. The second scenario is when a few applications, such as gas and oil, it is required to increase the reflectivity and bandwidth. Therefore, that plan results in having six test cases. All test cases have used the same optimization parameters to evaluate the performance parameters for all optimization methods, as listed in Table 1:

### 5.1. Testing Optimization Based on NSGA-II

**Figure 4:**
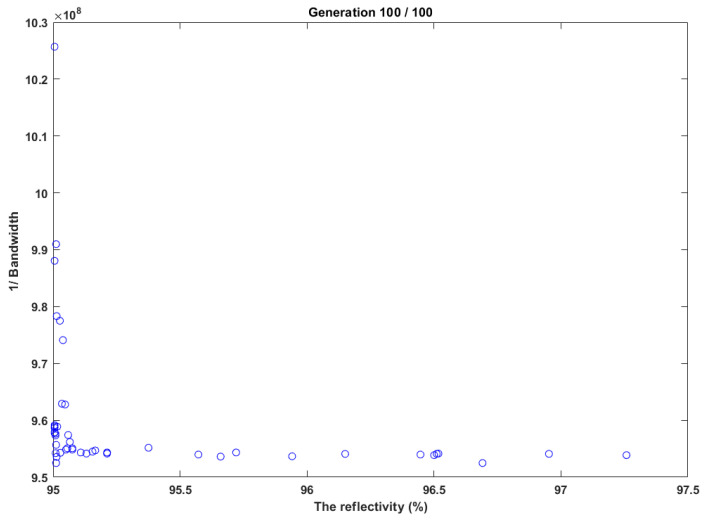
NSGA-II test case for high reflectivity and narrow bandwidth.

In this test case, NSGA has been tested, as shown in Figure 4. It determined the grating parameters that realize a high reflectivity of 95% and narrow bandwidth of less than 1 nm. Figure 4 shows the results of NSGA-II for optimizing the bandwidth and reflectivity to increase reflectivity and minimize bandwidth. The results demonstrated in Figure 4 clearly show that the Pareto front is determined successfully, the reflectivity was reported as 96.902%, and the bandwidth was 0.830319 nm. 

**Figure 5:**
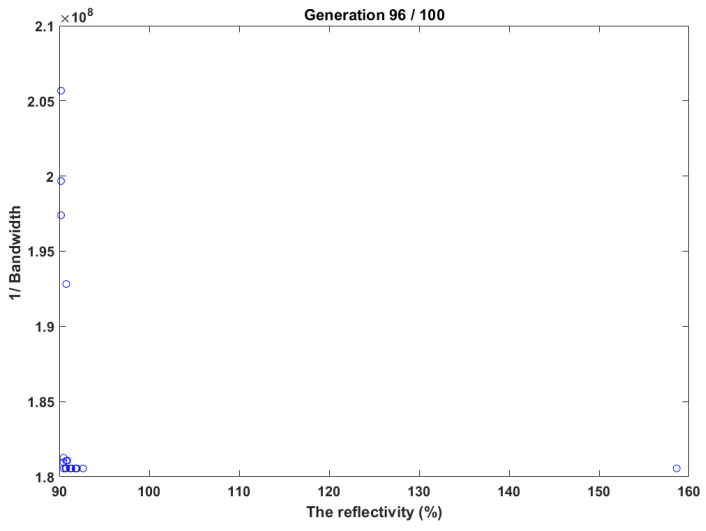
Testing of NSGA-II for increasing the reflectivity and increasing the bandwidth.

Figure 5 shows the results of NSGA-II for optimizing the bandwidth and reflectivity with a contradicting case in which the objective of increasing reflectivity and increasing the bandwidth is also. The results show that the Pareto front is determined successfully, and the reflectivity was reported to be 90.193%, and the bandwidth was 4.79425 nm.

### 5.2. Testing Optimization Based on RNSGA-II

**Figure 6:**
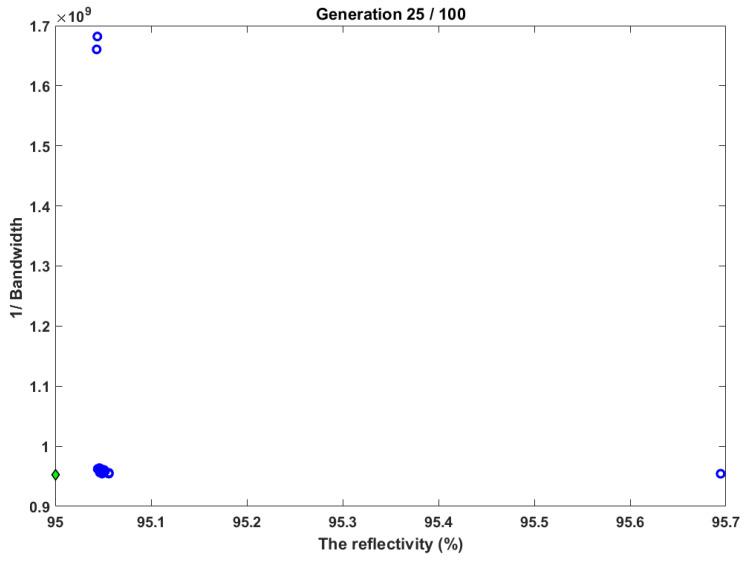
The testing of RNSGA-II for increasing the reflectivity and decreasing the bandwidth.

In this test case, the RNSGA-II is tested to show the merits of having a reference based on which the results will be influenced. Figure 6 shows the case res to maximize the reflectivity and minimize the bandwidth. It offers two merits of the usage of RNSGA-II, which are: collecting the closest to the reference point marked in green point. The reference point was set to 95% and 1.045 for reflectivity and bandwidth, respectively. Accordingly, the obtained reflectivity from that test was 95.01, and the bandwidth was 1.04775 nm. 

**Figure 7:**
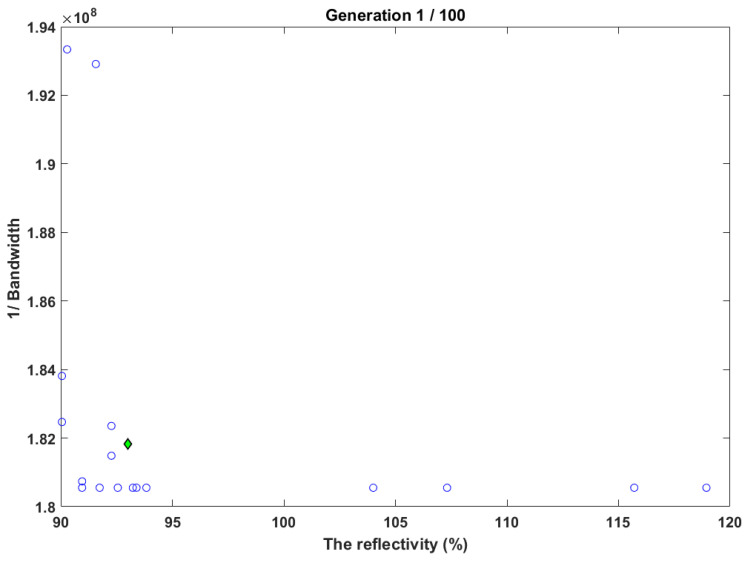
The testing RNSGA-II for increasing the reflectivity and increasing the bandwidth.

Similarly, the contradictory condition is tested with the RNSGA-II, as shown in Figure 7 depicts the results of increasing the bandwidth and the reflectivity. It shows that the Pareto front is obtained successfully, and the nearest values to the reference point set have been determined. The results were 4.95385 and 90.067% for the bandwidth and reflectivity, respectively. 

### 5.3. Testing Optimization Based on GA

**Figure 8:**
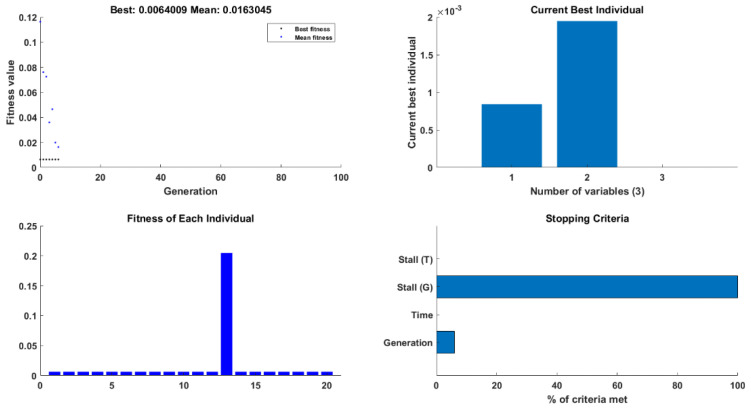
The testing GA for optimizing the reflectivity and bandwidth.

The GA has been selected as one of the most common optimization methods to be compared with the proposed methods. Figure 8 shows the results of the GA optimization test, including the fitness and convergence, cost function, and solution. Results were 40.169% and 2.93851 for reflectivity and bandwidth in the case of targeting reducing the bandwidth and increasing the reflectivity. Moreover, in the second scenario for increasing the reflectivity and bandwidth, the results were 5.53859 nm for the bandwidth and 55.151% for the reflectivity. 

### 5.4. Validating and Discussing the Results

For comparing and validating the proposed methods with traditional optimization methods, Table 2 compares the obtained results for all test cases, including GA, NSGA-II, and RNSGA-II. Numbers show clearly that the two proposed ways can successfully fetch the Pareto front and work in all scenarios determining the grating parameters for all sensing application needs even when there is a conflict between objectives. However, the GA could not determine the Pareto front. In addition, the NSGA-II showed higher reflectivity than that obtained by using the RNSGA-II. However, the opposite was true for bandwidth.

## 6. Validation and Comparison with Literature

**Figure 9:**
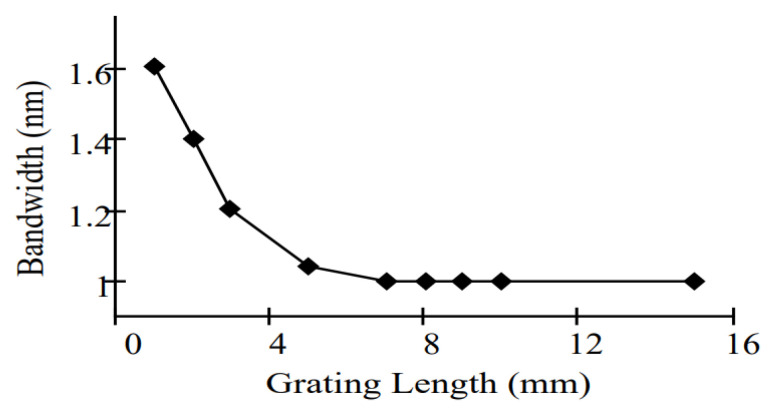
Relationship between FBG sensor bandwidth and grating length [15].

**Figure 10:**
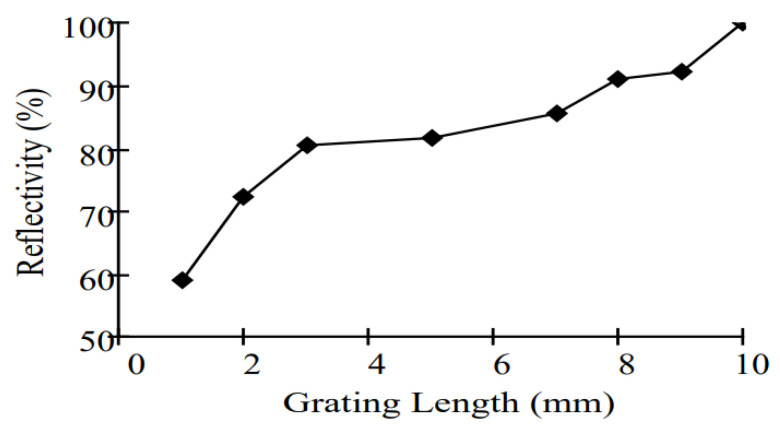
Relationship between FBG sensor reflectivity and grating length [15].

Many studies were found in the literature that contributed to determining the relation between the grating parameters such as the grating length and the change in reflective index and the reflectivity and bandwidth [15,16]. One of these studies has experimented with varying the grating length from 1 to 10 nm to determine a wider bandwidth to fulfill the requirements of oil and gas applications [15]. In that study, the authors kept varying the grating length and monitoring the change in bandwidth and reflectivity, as shown in Figure 9 and Figure 10. They offer a positive correlation between the reflectivity and grating length, while the correlation between the bandwidth and grating length is negative. That shows the problem for which this study proposed a solution. In addition, this method does not offer an optimization; instead, it determines the suitable value based on varying the grating length and remarking the equivalent bandwidth and reflectivity. By applying the same parameters to the proposed optimization methods, higher reflectivity has been obtained and can handle the two contradictory test cases. 

**Figure 11:**
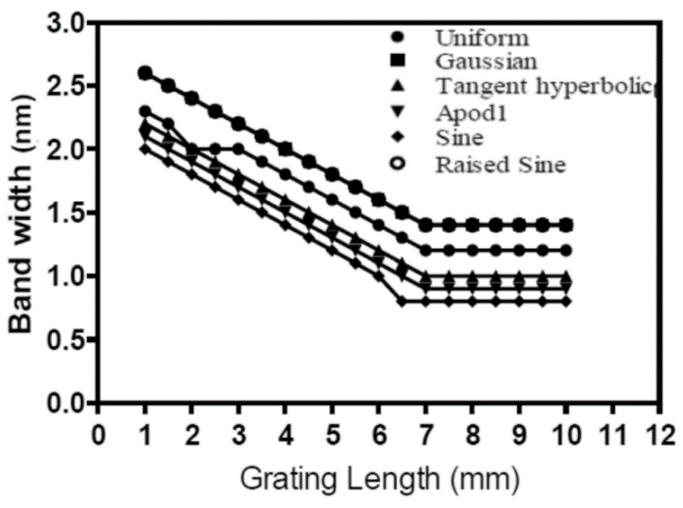
Relationship between FBG sensor bandwidth and grating length [16].

**Figure 12:**
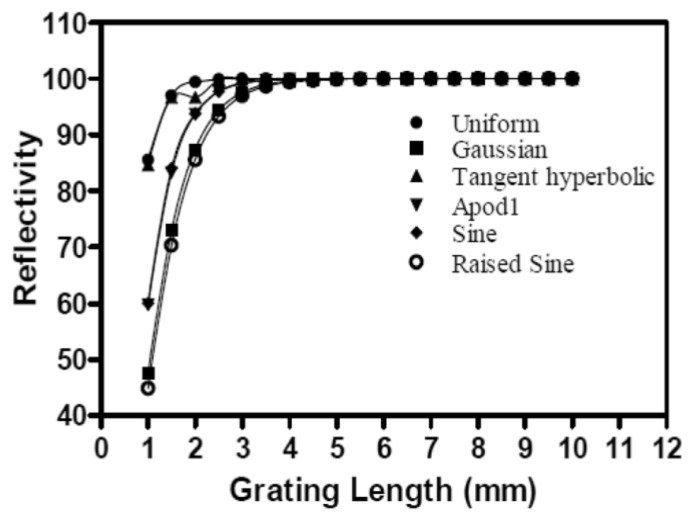
Relationship between FBG sensor reflectivity and grating length [16].

Similarly, another study tried to determine the least bandwidth and the highest reflectivity by changing the grating length and remarking on the bandwidth and reflectivity [16]. The study showed a negative correlation between the bandwidth and the grating length, as shown in Figure 11. Moreover, it shows a positive correlation between the grating length and the reflectivity, as depicted in Figure 12. However, the proposed method showed a complete optimization in that c in the contradicting test case. In addition, the current method shows higher reflectivity and narrower bandwidth. 

## 7. Conclusions

This research proposes two methods, NSGA-II and RNSGA-II. They apply Pareto optimization concepts for optimizing a multi-objective function that might have some conflicts due to the different needs of industrial applications. The two methods have been implemented and tested with different scenarios. In addition, one of the most common optimization methods, GA, has been chosen to validate the proposed methods. The two proposed ways successfully determined the Pareto front offering the decision makers the solutions of the whole set of solutions that provide the optimum value for reflectivity and bandwidth in two contradicting scenarios. One is when reflectivity and bandwidth must be maximized, such as in gas and oil applications. Moreover, another scenario is when it is required to maximize the reflectivity but decrease the bandwidth. Comparing the proposed methods, NSGA-II and RNSGA-II, with the GA, it is found that GA could not handle the contradiction in objectives for different applications’ requirements. In addition, it shows that the NSGA-II has the highest reflectivity, and the RNSGA-II showed the largest bandwidth. To sum up, having a tool that interprets the application requirements of bandwidth and reflectivity into the optimum manufacturing grating parameters can advance FBG sensing manufacturing and can be used as a tool for designing the best grating parameters for the FBG sensing industry.

## Figures and Tables

**Table 1:** The testing parameters of optimization.

Parameter	Values
Population size	20
Maximum generation	100
Number of objectives	2
Number of variables	3
Number of constraints	3
The radius of the core	4.50 μm
Core refractive index	1.449 μm
Cladding refractive index	1.444 μm
Center wavelength	1.55 μm

**Table 2:** Comparison between NSGA-II, RNSGA-II, and GA.

Method	Modulation Index (mm)	Grating Length (mm)	Grating Period (nm)	Bandwidth (nm)	Reflectivity (%)
Test case I: maximize reflectivity and minimize bandwidth					
NSGA-II	0.0007	5.968	0.1	0.830319	96.902
R-NSGA-II	0.0008	6.005	0.2	1.04775	95.01
GA	0.0003	1.815	0.5	2.93851	40.169
Test case II: maximize reflectivity and maximize bandwidth					
NSGA-II	0.0006	2.8	1.01	4.79425	90.193
R-NSGA-II	0.0004	3.3	0.98	4.95385	90.067
GA	0.0002	10	10.01	5.53859	55.151

## Data Availability

Not applicable.
